# Amphetamine use and Parkinson’s disease: integration of artificial intelligence prediction, clinical corroboration, and mechanism of action analyses

**DOI:** 10.1371/journal.pone.0323761

**Published:** 2025-05-20

**Authors:** Maria P. Gorenflo, Zhenxiang Gao, Pamela B. Davis, David Kaelber, Rong Xu

**Affiliations:** 1 Center for Artificial Intelligence in Drug Discovery, School of Medicine, Case Western Reserve University, Cleveland, Ohio, United States of America; 2 Cleveland Clinic Lerner College of Medicine, Case Western Reserve University, Cleveland, Ohio, United States of America; 3 Center for Community Health Integration, Case Western Reserve University School of Medicine, Euclid Avenue, Cleveland, Ohio, United States of America; 4 Center for Clinical Informatics Research and Education, The MetroHealth System, MetroHealth Drive, Cleveland, Ohio, United States of America; Ladoke Akintola University of Technology Teaching Hospital: LAUTECH Teaching Hospital, NIGERIA

## Abstract

Parkinson’s disease (PD) is an increasingly prevalent neurologic condition for which symptomatic, but not preventative, treatment is available. Drug repurposing is an innovate drug discovery method that uncovers existing therapeutics to treat or prevent conditions for which they are not currently indicated, a method that could be applied to incurable diseases such as PD. A knowledge graph artificial intelligence prediction system was used to select potential drugs that could be used to treat or prevent PD, and amphetamine was identified as the strongest candidate. Retrospective cohort analysis on a large, electronic health record database of deidentified patients with attention deficit hyperactive disorder (the main diagnosis for which amphetamine is prescribed) revealed a significantly reduced hazard of developing PD in patients prescribed amphetamine versus patients not prescribed amphetamine at 2, 4, and 6 years: Hazard Ratio (95% Confidence Interval) = 0.59 (0.36, 0.98), 0.63 (0.42, 0.93), and 0.55 (0.38, 0.79). Pathway enrichment analysis confirmed that amphetamine targets many of the biochemical processes implicated in PD, such as dopaminergic synapses and neurodegeneration. Together, these observational findings suggest that therapeutic and legal amphetamine use may reduce the risk of developing PD, in contrast to previous work that found the inverse relationship in patients using amphetamine recreationally.

## Introduction

Parkinson’s disease (PD) is a common neurodegenerative condition in the United States, affecting over one million Americans as of 2019 [1]. It involves degeneration of substantia nigra dopaminergic neurons, leading to tremor, bradykinesia, rigidity, and psychiatric and autonomic disturbances that profoundly impair daily life [2]. Current therapies for PD can effectively slow its advance, such as carbidopa/levodopa to increase intracerebral dopamine levels and deep-brain stimulation to modulate electrical activity within the basal ganglia [3]. However, it is still an incurable disease, and considering its high prevalence and disease burden in the United States, there is a need for further strategies to address and mitigate PD. One option would be to delay or prevent the development of PD, such that those who are at-risk or with prodromal presentation of the disease could be targeted to reduce the risk of developing it [4]. There is observational evidence that physical activity, diet, chemical exposures, and certain drugs all modulate the risk of developing PD [5–7]. However, there is an overall paucity of research focusing on drugs that could be prescribed to reduce the risk of developing PD in high-risk individuals.

Drug repurposing is a process that involves identifying new indications for approved drugs, bypassing the traditional time- and investment-consuming drug discovery process [8]. In recent years, the application of artificial intelligence (AI) technology has further accelerated the drug repurposing process. AI algorithms analyze medical and biochemical data to uncover new indications for existing drugs, further accelerating and de-risking the drug development process [9,10]. While these methodologies have indeed provided a wealth of potential for drug repurposing, it is important to note that numerous drug candidates, initially promising, have unfortunately failed in subsequent clinical trial testing due to insufficient efficacy [8]. This highlights the necessity of additional rounds of rigorous vetting before any candidate drug is propelled into clinical trial phases. Vetting can be significantly enhanced by the integration of data from electronic health records (EHRs). EHRs provide a rich source of real-world data that can offer invaluable insights into patient health histories, disease progression, and treatment responses [11]. This information becomes even more crucial in the context of drug repurposing, where the focus is on finding new indications for existing drugs. Access to multiple EHRs is especially important, as the study population must include patients who have been prescribed the candidate drugs and who may develop the disease of interest. This allows researchers to investigate potential correlations between the use of the drug and the progression or regression of the disease of interest. Integration of EHRs provides a broader and more accurate understanding of the behavior and performance of drugs in diverse patient populations, thus enabling a more precise assessment of a drug’s potential for repurposing.

This study utilizes a drug repurposing strategy introduced in our earlier work [12–15] that integrates a knowledge graph AI prediction system, clinical corroboration, and mechanism of action analysis to uncover a Food and Drug Administration (FDA)-approved drug that could be used to delay or prevent the onset of PD.

## Methods

### Knowledge graph prediction system

Drug repurposing for PD began with our AI-based drug discovery model, Knowledge Graph (KG)-Predict [15–17]. A knowledge graph was constructed by integrating interactions between drugs, genes, diseases, and biomedical ontologies from various public biomedical datasets. Biomedical ontologies for the knowledge graph include Human Phenotype Ontology [18], Mondo Disease Ontology [19], Gene Ontology [20], and Entrez Gene [21]. The following biomedical knowledge bases for entity and relation data were also included: DrugBank [22], side effect knowledgebases, Drug Central [23], the DisGeNET knowledgebase [24], protein-protein interaction networks [25], and the Reactome Pathway Database [26]. After knowledge integration and biomedical entity term normalization, the knowledge graph contained a total of 108,146 entities of 9 types and 19 relation types within 9 kinds of entity pairs (S1 Table).

**Fig 1:**
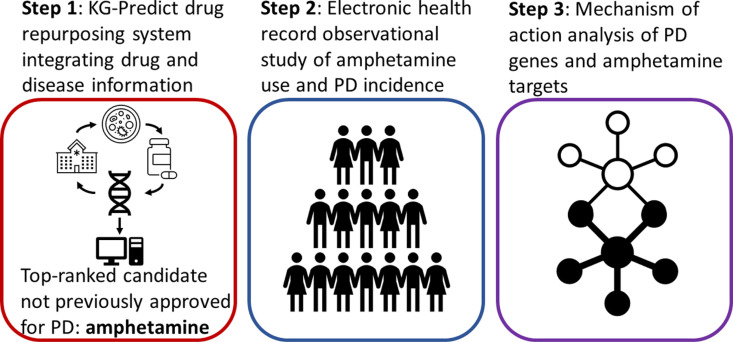
Overall study design.

Based on the knowledge graph, KG-Predict identified potential repositioned drugs for PD by establishing heterogeneous information around drugs, genes, and diseases. The model first encoded entities, such as drugs, diseases, and genes, along with their intricate interactions within the knowledge graph, into vector spaces. This encoding captured hidden patterns and structural relationships within the graph. In the subsequent link prediction phase, KG-Predict utilized these vector representations to uncover previously unknown connections between drugs and the target disease (PD in this study). Each drug is assigned a composite score reflecting its interactions with PD-associated targets, pathways, phenotypic annotations, and network connectivity. Consequently, a drug demonstrating more significant connectivity within PD will receive a higher rank than one with weaker or fewer connections. Notably, all drug-PD interactions were removed from the knowledge graph during training, ensuring KG-Predict identified potential candidates based on indirect relationships and existing knowledge, thereby preventing data leakage and overestimation of its inference capabilities. The top-ranked drug for PD from this KG-Predict system that was not already approved to treat the disease is amphetamine, so this was the drug of focus for the analysis (Fig 1).

## Clinical corroboration of amphetamine

### Data sources

TriNetX Analytics was used to characterize the relationship between amphetamine and PD on the population level. TriNetX is a cloud-based EHR platform with up-to-date data from over 90 million unique American patients with diverse racial, ethnic, and socioeconomic backgrounds from all 50 states (US Collaborative Network). TriNetX reports population-level data without protected health information; therefore, the MetroHealth System, Cleveland, Institutional Review Board has determined that this utilization of TriNetX is not Human Subject Research and is therefore exempt from review.

### Study population

**Fig 2:**
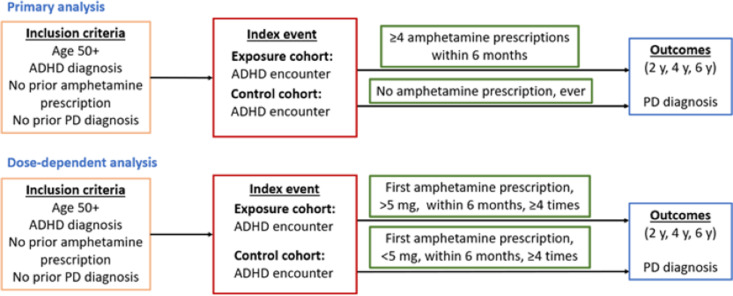
Retrospective cohort analysis study design.

As the main indication for amphetamine prescription is attention deficit hyperactivity disorder (ADHD) [27], and PD is most often diagnosed after age 50 [28], the study population is comprised of patients with a diagnosis of ADHD over the age of 50. Patients with a PD diagnosis or amphetamine prescription prior to the index event were excluded from analysis (Fig 2). The index event of the exposure and control cohorts is a healthcare encounter for ADHD. The exposure cohort consists of patients with at least four amphetamine prescriptions within six months of this ADHD encounter, as amphetamine’s status as a controlled substance necessitates its frequent prescription. The control cohort consists of patients with no amphetamine prescription ever. The outcome of interest is PD diagnosis at 2, 4, or 6 years after the index event (Fig 2).

### Statistical analysis

The following analyses were run on TriNetX built-in analytic tools. The cohorts were propensity-score matched for demographic factors (age at index event, sex, race, and ethnicity), as well as diagnoses, drug prescriptions, and lab values associated with increased or reduced risk of developing PD (nicotine dependence, alcohol related disorders, injuries to the head, diabetes mellitus, hyperuricemia, and melanoma; prescription of calcium channel blockers or estrogen; hemoglobin A1C value) [4]. The TriNetX, International Classification of Diseases (ICD)-10, and Veterans Affairs (VA) Formulary codes for the demographics, diagnoses, and drugs used to create the exposure and control cohorts and match between them are available in Table 1.

**Table 1:** Patient characteristics.

TriNetX, ICD-10, or VA Formulary Code	Characteristic	Exposure Cohort Before Matching (n = 14,042)	Control Cohort Before Matching (n = 64,037)	SMD	Exposure Cohort After Matching (n = 13,992)	Control Cohort After Matching (n = 13,992)	SMD
Demographics							
AI	Age at Index	56.69 (6.19)	58.47 (7.44)	0.26	56.69 (6.19)	56.71 (6.30)	<0.01
M	Male	4 870 (34.783%)	27 473 (43.184%)	0.17	4 870 (34.806%)	4 989 (35.656%)	0.02
F	Female	8 746 (62.467%)	34 523 (54.266%)	0.17	8 737 (62.443%)	8 642 (61.764%)	0.01
2106-3	White	12 437 (88.829%)	53 758 (84.501%)	0.13	12 428 (88.822%)	12 508 (89.394%)	0.02
2054-5	Black or African American	399 (2.85%)	3 108 (4.885%)	0.11	399 (2.852%)	406 (2.902%)	<0.01
2131-1	Unknown Race	1 066 (7.614%)	6 224 (9.783%)	0.08	1 066 (7.619%)	994 (7.104%)	0.02
2135-2	Hispanic or Latino	409 (2.921%)	1 671 (2.627%)	0.02	409 (2.923%)	379 (2.709%)	0.01
2186-5	Not Hispanic or Latino	11 642 (83.151%)	48 684 (76.526%)	0.17	11 635 (83.155%)	11 778 (84.177%)	0.03
UN	Unknown Ethnicity	1 950 (13.928%)	13 263 (20.848%)	0.18	1 948 (13.922%)	1 835 (13.115%)	0.02
Diagnoses							
F17	Nicotine dependence	2 845 (20.32%)	7 596 (11.94%)	0.23	2 836 (20.269%)	2 844 (20.326%)	<0.01
E08-E13	Diabetes mellitus	2 051 (14.649%)	7 949 (12.495%)	0.06	2 048 (14.637%)	2 115 (15.116%)	0.01
S00-S09.	Injuries to the head	1 943 (13.878%)	5 709 (8.974%)	0.15	1 940 (13.865%)	1 916 (13.694%)	<0.01
F10	Alcohol related disorders	1 315 (9.392%)	3 749 (5.893%)	0.13	1 310 (9.362%)	1 321 (9.441%)	<0.01
M10	Gout	401 (2.864%)	1 199 (1.885%)	0.06	400 (2.859%)	381 (2.723%)	0.01
C43	Malignant melanoma	144 (1.028%)	452 (0.71%)	0.03	144 (1.029%)	130 (0.929%)	0.01
Lab values							
9037	Hemoglobin A1c < 5.7%	3 165 (22.606%)	7 597 (11.942%)	0.28	3 156 (22.556%)	3 209 (22.935%)	0.01
9037	Hemoglobin A1c 5.7–6.4%	2 292 (16.37%)	5 985 (9.408%)	0.21	2 285 (16.331%)	2 244 (16.038%)	0.01
9037	Hemoglobin A1c > 6.4%	1 226 (8.757%)	4 230 (6.649%)	0.08	1 225 (8.755%)	1 278 (9.134%)	0.01
Prescriptions							
CV200	Calcium channel blockers	2 021 (14.435%)	6 446 (10.132%)	0.13	2 016 (14.408%)	2 126 (15.194%)	0.02
HS300	Estrogens	1 923 (13.735%)	3 383 (5.318%)	0.29	1 914 (13.679%)	1 873 (13.386%)	0.01

A nearest neighbor greedy matching algorithm was used to conduct 1:1 propensity score matching with a caliper of 0.25 standard deviations. A Kaplan-Meier survival analysis [29] was conducted on the matched cohorts to estimate the probability of developing PD at 2, 4, and 6 years after the index event ADHD encounter in both the exposure and control cohorts. Subsequently, the cohorts were compared using Cox’s proportional hazards model, with the proportional hazard assumption being tested with the generalized Schoenfeld approach. Hazard ratios (HR) and 95% confidence intervals (CI) were generated to quantify the relative hazard of PD diagnosis based on comparison of time to event rates (Fig 1). Separate subgroup analyses were performed in cohorts stratified by gender, as estrogen is a potential effect modifier in the relationship between amphetamine exposure and dopamine signaling in the brain [30–34]. There was an additional analysis run by comparing the hazard of developing PD in patients prescribed greater than 5 milligrams (mg) amphetamine versus those prescribed less than 5 mg as the control group to determine if a dose-dependent relationship between amphetamine and PD incidence exists (Fig 2).

### Mechanism of action analysis

Thirty-five genes associated with PD were obtained from DisGeNet [24], a comprehensive database of disease-gene associations. Sixty-three amphetamine-associated genes were collected from the Search Tool for Interactions of Chemicals (STITCH) database [35]. The Kyoto Encyclopedia of Genes and Genomes (KEGG) database [36] provided pathway information to perform pathway enrichment analysis. Genetic pathways from KEGG for each PD-associated gene were obtained, using P < 0.05 as the threshold. The same method was applied to identify genetic pathways significantly enriched for amphetamine. The PD- and amphetamine-associated pathways were intersected to identify the pathways involved in both PD pathophysiology and amphetamine use (Fig 1).

## Results

### Top candidate drug for PD identified by KG-Predict

To assess the model’s ability to identify potential drug candidates for PD treatment, all drug-PD interactions were excluded from the knowledge graph during training. This step was taken to prevent data leakage and ensure a more accurate evaluation of the model’s predictive performance, avoiding overestimation of its ability to infer novel drug-PD associations. The top 30 drugs generated by KG-Predict to treat or prevent PD are listed in the S2 Table. Among these, 26 have been approved for the treatment of PD, and two have been tested in clinical trials for PD, results which validate KG-Predict’s ability to identify drugs for this disease. Amphetamine was ranked first among non-approved drugs for PD. Follow-up retrospective cohort studies using patients’ EHR data were conducted for amphetamine.

### Amphetamine prescription is associated with reduced hazard of PD diagnosis

**Fig 3:**
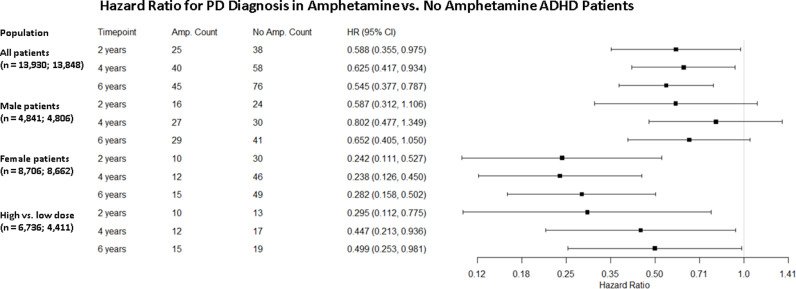
Retrospective cohort analysis results.

On the TriNetX US Collaborative Network, there were 78,079 patients with an ADHD diagnosis over the age of 50 when the analysis was conducted in January 2023. This includes 14,042 who received at least four prescriptions of amphetamine in the six months after their ADHD encounter, and 64,037 with no amphetamine prescription ever (Table 1). After matching and excluding those with a prior PD diagnosis, there were 13,930 and 13,848 patients in the exposure and control cohorts, respectively. In the exposure cohort, the average age is 56.7 years; 34.8% of patients are male; and 88.8% of the cohort is white. In the control cohort, the average age is 56.7 years; 35.7% of patients are male; and 89.4% of the cohort is white (Table 1). There is a significantly reduced hazard of PD diagnosis in the exposure versus control cohorts at 2, 4, and 6 years: HR = 0.59 (95% CI: 0.36, 0.98), 0.63 (95% CI: 0.42, 0.93), and 0.55 (95% CI: 0.38, 0.79) (Fig 3).

In the subgroup analysis of male patients, there is no significant difference in the hazard of PD diagnosis between the exposure and control cohorts at any of the three timepoints: HR = 0.59 (95% CI: 0.31, 1.11), 0.80 (95% CI: 0.48, 1.35), and 0.65 (95% CI: 0.41, 1.05). In the subgroup analysis of female patients, on the other hand, there is a significantly reduced hazard of PD diagnosis in the exposure cohort, of a greater magnitude than that seen in the overall cohorts: HR = 0.24 (95% CI: 0.11, 0.53), 0.24 (95% CI: 0.13, 0.45), and 0.28 (95% CI: 0.16, 0.50). In the dose-relationship subgroup analysis, patients prescribed greater than 5 mg of amphetamine displayed reduced risk of PD diagnosis at all three timepoints compared to those prescribed less than 5 mg of amphetamine: HR = 0.30 (95% CI: 0.11, 0.78); 0.45 (95% CI: 0.21, 0.94); 0.50 (95% CI: 0.25, 0.98) (Fig 3).

### Amphetamine is associated with many genes and pathways implicated in PD

To investigate potential biochemical mechanisms linking amphetamine use with PD pathophysiology, we performed pathway enrichment analysis on amphetamine and PD related genes. Thirty-five PD-associated genes were identified. Amphetamine targets 63 genes, eight of which are associated with PD: MAOB, DRD2, SNCA, SLC18A2, DDC, DRD1, TH, and SLC6A3 (see the complete list of genes in the S3 Table). Twelve genetic pathways are significantly enriched for the PD-associated genes, 22 are significantly enriched for the amphetamine-associated genes, and among these 11 are significantly enriched for both (see the complete list of pathways in the S4 Table). Shared pathways for PD and amphetamine include PD, amphetamine addiction, dopaminergic synapse, and pathways of neurodegeneration - multiple diseases (Table 2).

**Table 2:** Number of significantly enriched pathways for amphetamine, number of significantly enriched pathways shared between amphetamine and PD, and list of shared pathways.

Candidate	Total	Shared	Pathways
Amphetamine	22	11	Parkinson disease
			Cocaine addiction
			Amphetamine addiction
			Dopaminergic synapse
			Pathways of neurodegeneration - multiple diseases
			Alcoholism
			Tyrosine metabolism
			Phenylalanine metabolism
			Serotonergic synapse
			Tryptophan metabolism
			Synaptic vesicle cycle

## Discussion

This analysis integrated an AI-based drug discovery model, real-world population-level data in TriNetX, and pathway enrichment analysis to repurpose FDA-approved drugs for delaying or preventing PD onset. To our knowledge this is the first study to utilize an integrated knowledge graph prediction system to identify a drug that could be repurposed for PD. Amphetamine was ranked as the top candidate among non-approved PD drugs for clinical corroboration via EHR evaluation. The results suggest that amphetamine may be associated with reduced risk of PD diagnosis in patients with ADHD, especially women. Amphetamine functions by increasing monoamine release, especially dopamine, into the post-synaptic cleft throughout the brain [37]. Considering that PD is characterized by destruction of dopaminergic neurons, perhaps the augmentation of dopamine release triggered by amphetamine can delay the onset of PD. This hypothesis is bolstered by our pathway analysis, which revealed that pathways involving dopaminergic synapses are implicated in both amphetamine and PD. This association is stronger amongst female compared to male patients, perhaps due to the effect that estrogen has on amphetamine-stimulated dopamine signaling in the brain [30–34]. There is also some preliminary evidence from this analysis that amphetamine exerts a causal effect on developing PD: one is the inverse dose-dependent relationship between amphetamine prescription and PD diagnosis, and the other is the pathway analysis that identified eight shared genes and 11 shared biochemical pathways between amphetamine and PD. However, causality cannot be concluded from the evidence presented here.

Another important consideration is the relationship between ADHD and PD. Patients with ADHD were selected as the study population because they are the major recipients of amphetamine prescriptions. However, the pathophysiology of ADHD involves imbalances of dopamine signaling in the brain, including the basal ganglia, representing some overlap with the etiology of PD [38]; there is contradicting evidence pertaining to the presence of a causal relationship between the two conditions [39,40]. If indeed a causal relationship between the two exists, then amphetamine use may target pathways involved in both conditions. Should ADHD correspond to increased PD risk, this should not impact the interpretation of our study, as all patients in the exposure and control cohort have an ADHD diagnosis. Even if ADHD severity correlates to PD risk, then one would expect that higher-dose amphetamine patients would have an increased PD risk (as the increased dose may be in response to greater disease severity), but the opposite is observed. Therefore, while the interconnection between amphetamine, ADHD, and PD is an intriguing one that is important to consider and better understand, it does not alter the conclusions of this analysis.

Interestingly, several existing retrospective clinical studies have revealed the opposite correlation to that described here (amphetamine use associated with increased PD incidence), but these were in the context of patients with recreational amphetamine use or amphetamine use disorder [41,42]. Importantly, excess and recreational amphetamine use causes excitotoxicity and oxidative damage in the central nervous system [43], both phenomena linked to the development of PD [44,45]. Therefore, it is possible that controlled, prescribed, and therapeutic doses of amphetamine reduce PD risk or delay its onset by increasing dopaminergic activity in the brain, whereas excessive levels increase PD risk through mechanisms of central nervous system toxicity.

There are several important limitations inherent to our study. Firstly, the EHR data available on TriNetX are not a random sample of the entire country; therefore, the results presented here may not be generalizable to the entire United States population. For example, in this study over 2/3 of ADHD patients are females, but overall there are more males than females with ADHD [46]. Secondly, it is also possible that patients have diagnoses not captured in their EHR data in TriNetX; for example, some patients in the control cohort may have indeed received an amphetamine prescription in another healthcare system. This compromises the internal validity of the results. Thirdly, despite utilizing propensity score matching to improve the balance of covariate distributions and mitigate the impact of numerous confounders, the inherent observational nature of the study design may still result in the omission of certain unobserved and unmeasured confounders. This could potentially exacerbate data imbalance and introduce bias. Finally, the current list of genes and pathways underlying the etiology of PD is likely incomplete. However, as new genes and pathways associated with PD are discovered in the future, they may enhance the identification of potential drugs for treating PD.

Overall, the results presented here reveal the potential for amphetamine prescription to delay or prevent the onset of PD. Future work should focus on cellular studies investigating the mechanism of action of the relationship between amphetamines, ADHD, and PD; characterizing the levels at which amphetamine may reduce versus increase PD risk; distinguishing the effects of racemic amphetamine versus dextroamphetamine on PD risk; and conducting clinical trials to determine if a truly causal and clinically significant relationship exists.

## Supporting information

S1 Table Statistics of nodes and interactions in the knowledge graph. (DOCX)

S2 Table Top 30-ranked drug candidates associated with PD. (DOCX)

S3 Table The complete list of PD- and amphetamine-associated genes. (DOCX)

S4 Table The full list of enriched pathways for PD and amphetamine. (DOCX)
