# Supplementary material for: Amphetamine use and Parkinson’s disease: integration of artificial intelligence prediction, clinical corroboration, and mechanism of action analyses
Source: PLoS One. 2025 May 20;20(5):e0323761. doi: 10.1371/journal.pone.0323761 (PMC12091834; doi:10.1371/journal.pone.0323761)
Supplement: S1 Table — (DOCX) [file pone.0323761.s001.docx]

**1. S1 Table.** Statistics of nodes and interactions in the knowledge graph.

| **Data Source** | **Interaction Type** | **Interaction Number** | **Entity Type** | **Entity Number** |
| --- | --- | --- | --- | --- |
| DrugBank | drug_gene | 25,468 | drug | 6,282 |
|  |  |  | gene | 3,094 |
| Sider | drug_effect | 64,784 | drug | 1,137 |
|  |  |  | side effect | 990 |
| Drug Central | indication  (Drug-Disease) | 9,388 | drug | 1,801 |
|  |  |  | disease | 1,363 |
|  | contraindication | 30,675 | drug | 1,263 |
|  |  |  | disease | 1,195 |
| HPO | disease_phenotype_positive | 150,317 | disease | 6,744 |
|  |  |  | human phenotype | 9,404 |
|  | disease_phenotype_negative | 1,193 | disease | 686 |
|  |  |  | human phenotype | 579 |
|  | phenotype_phenotype | 18,736 | human phenotype | 14,885 |
|  |  |  | human phenotype | 14,885 |
| Mondo Disease Ontology | disease_disease  (parent-child) | 32,194 | disease | 17,008 |
|  |  |  | disease | 17,008 |
| DisGeNET | disease_gene | 80,411 | disease | 5,593 |
|  |  |  | gene | 9,080 |
| HPO | phenotype_gene | 3330 | human phenotype | 356 |
|  |  |  | gene | 1,621 |
| PPI | gene_gene | 321,075 | gene | 18,354 |
|  |  |  | gene | 18,354 |
| Gene Ontology/  Entrez Gene | bioprocess_gene | 144,805 | biological processes | 12,459 |
|  |  |  | gene | 18,650 |
|  | bioprocess_bioprocess | 52,886 | biological processes | 28,642 |
|  |  |  | biological processes | 28,642 |
|  | cellcomp_gene | 83,402 | cellular components | 1,756 |
|  |  |  | gene | 19,363 |
|  | cellcomp_cellcomp | 4,845 | cellular components | 4,176 |
|  |  |  | cellular components | 4,176 |
|  | molfunc_gene | 69,530 | molecular functions | 4,369 |
|  |  |  | gene | 18,148 |
|  | molfunc_molfunc | 13,574 | molecular functions | 11,169 |
|  |  |  | molecular functions | 11,169 |
| Reactome pathway  database | pathway_gene | 42,592 | pathway | 2,006 |
|  |  |  | gene | 10,849 |
|  | pathway_pathway | 2,523 | pathway | 2,498 |
|  |  |  | pathway | 2,498 |
|  | Total interactions | 1,151,728 | Total entities | 108,146 |
