# Supplementary material for: Amphetamine use and Parkinson’s disease: integration of artificial intelligence prediction, clinical corroboration, and mechanism of action analyses
Source: PLoS One. 2025 May 20;20(5):e0323761. doi: 10.1371/journal.pone.0323761 (PMC12091834; doi:10.1371/journal.pone.0323761)
Supplement: S2 Table — (DOCX) [file pone.0323761.s002.docx]

**2. S2 Table.** Top 30-ranked drug candidates associated with PD.

| **No** | **Drug** | **Indication** | **Evidence** |
| --- | --- | --- | --- |
| 1 | Levodopa | Levodopa on its own is formulated as an oral inhalation powder indicated for intermittent treatment of off episodes in Parkinson's patients who are already being treated with carbidopa and levodopa. Levodopa is most commonly formulated as an oral tablet with a peripheral dopa decarboxylase inhibitor indicated for treatment of Parkinson's disease, post-encephalitic parkinsonism, and symptomatic parkinsonism following carbon monoxide intoxication or manganese intoxication. | FDA-Approved |
| 2 | Carbidopa | Carbidopa is indicated with for the treatment of symptoms of idiopathic Parkinson disease, postencephalitic parkinsonism and symptomatic parkinsonism followed by carbon monoxide or manganese intoxication. | FDA-Approved |
| 3 | Biperiden | For use as an adjunct in the therapy of all forms of parkinsonism and control of extrapyramidal disorders secondary to neuroleptic drug therapy. | FDA-Approved |
| 4 | Amantadine | For the chemoprophylaxis, prophylaxis, and treatment of signs and symptoms of infection caused by various strains of influenza A virus. Also for the treatment of parkinsonism and drug-induced extrapyramidal reactions. | FDA-Approved |
| 5 | Trihexyphenidyl | Indicated for the treatment of parkinson's disease and extrapyramidal reactions caused by drugs. | FDA-Approved |
| 6 | Benzatropine | Benztropine is indicated to be used as an adjunct in the therapy of all forms of parkinsonism. It can also be used for the control of extrapyramidal disorders due to neuroleptic drugs. | FDA-Approved |
| 7 | Entacapone | Used as an adjunct to levodopa / carbidopa in the symptomatic treatment of patients with idiopathic Parkinson's Disease who experience the signs and symptoms of end-of-dose "wearing-off". | FDA-Approved |
| 8 | Procyclidine | For the treatment of all forms of Parkinson's Disease, as well as control of extrapyramidal reactions induced by antipsychotic agents. | FDA-Approved |
| 9 | Pimavanserin | Investigated for use/treatment in neurologic disorders, parkinson's disease, psychosis, schizophrenia and schizoaffective disorders, and sleep disorders. | FDA-Approved |
| 10 | Cycrimine | For treatment and management of Parkinson's disease. | FDA-Approved |
| 11 | Ropinirole | For the treatment of the signs and symptoms of Parkinson's disease and for the treatment of primary moderate-severe restless legs syndrome. | FDA-Approved |
| 12 | Droxidopa | For treatment of neurogenic orthostatic hypotension (NOH) associated with various disorders including Multiple System Atrophy, Familial Amyloid Polyneuropathy, hemodialysis induced hypotension and Parkinson's Disease. Also investigated for use/treatment in neurologic disorders, nephropathy, blood (blood forming organ disorders, unspecified), and dizzy/fainting spells. | FDA-Approved |
| 13 | Apomorphine | Apomorphine is indicated to treat acute, intermittent treatment of hypomobility, off episodes associated with advanced Parkinson's disease. | FDA-Approved |
| 14 | Selegiline | Monotherapy for initial treatment of Parkinson's disease, as well as an adjunct therapy in patients with a decreased response to levodopa/carbadopa. Also used for the palliative treatment of mild to moderate Alzheimer's disease and at higher doses, for the treatment of depression. | FDA-Approved |
| 15 | Rasagiline | For the treatment of the signs and symptoms of idiopathic Parkinsons disease as initial monotherapy and as adjunct therapy to levodopa. | FDA-Approved |
| 16 | Rotigotine | For use/treatment in neurologic disorders and parkinson's disease as well as moderate-to-severe primary Restless Legs Syndrome. | FDA-Approved |
| 17 | Safinamide | Safinamide is indicated as an add-on treatment to levodopa with or without other medicines for Parkinson‚Äôs disease | FDA-Approved |
| 18 | Pramipexole | This drug is indicated for the symptomatic treatment of Parkinson‚Äôs disease. This drug can be administered as monotherapy or in conjunction with levodopa. It is also indicated for symptomatic treatment of moderate to severe primary Restless Legs Syndrome (RLS). | FDA-Approved |
| 19 | Istradefylline | Istradefylline is indicated in adjunct to levodopa and carbidopa in the treatment of Parkinson's disease. | FDA-Approved |
| 20 | Opicapone | Opicapone is approved as an adjunctive therapy in adults with Parkinson disease and end-of-dose motor fluctuations whose symptoms cannot be stabilized on levodopa/dopa decarboxylase inhibitor combinations. | FDA-Approved |
| 21 | Tolcapone | Used as an adjunct to levodopa/carbidopa therapy for the symptomatic treatment of Parkinson's Disease. This drug is generally reserved for patients with parkinsonian syndrome receiving levodopa/carbidopa who are experiencing symptom fluctuations and are not responding adequately to or are not candidates for other adjunctive therapies. | FDA-Approved |
| 22 | Bromocriptine | For the treatment of galactorrhea due to hyperprolactinemia, prolactin-dependent menstrual disorders and infertility, prolactin-secreting adenomas, prolactin-dependent male hypogonadism, as adjunct therapy to surgery or radiotherapy for acromegaly or as monotherapy is special cases, as monotherapy in early Parksinsonian Syndrome or as an adjunct with levodopa in advanced cases with motor complications. Bromocriptine has also been used off-label to treat restless legs syndrome and neuroleptic malignant syndrome. | FDA-Approved |
| 23 | Piribedil | Piribedil is a dopamine agonist used with or without levodopa in the treatment of Parkinson's disease. | FDA-Approved |
| 24 | Melevodopa | Melevodopa is indicated in combination with carbidopa for the treatment of Parkinson's disease. | FDA-Approved |
| 25 | Pergolide | Indicated as adjunctive treatment to levodopa/carbidopa in the management of the signs and symptoms of Parkinson's disease. It was withdrawn from the US and Canadian markets in 2007 due to an increased risk of cardiac valvulopathy. | FDA-Approved |
| 26 | Metixene | Used for the symptomatic treatment of parkinsonism. | FDA-Approved |
| 27 | Amphetamine | Amphetamine is indicated for the treatment of attention-deficit/hyperactivity disorders (ADHD) as well as for the treatment of central nervous system disorders such as narcolepsy. |  |
| 28 | Atomoxetine | Atomoxetine is indicated for the treatment of attention deficit hyperactivity disorder (ADHD) in children and adults. | NCT01738191, NCT00286949, NCT02879136, NCT00304161 |
| 29 | Epicriptine | Epicriptine is a nootropic indicated in individuals over sixty who manifest signs and symptoms of an idiopathic decline in mental capacity. |  |
| 30 | Clozapine | For use in patients with treatment-resistant schizophrenia. | NCT00004826 |

FDA: evidence from the Food and Drug Administration ([www.accessdata.fda.gov](http://www.accessdata.fda.gov)).

NCT: evidence from clinical trials ([www.clinicaltrials.gov](http://www.clinicaltrials.gov)).
