# Supplementary material for: Amphetamine use and Parkinson’s disease: integration of artificial intelligence prediction, clinical corroboration, and mechanism of action analyses
Source: PLoS One. 2025 May 20;20(5):e0323761. doi: 10.1371/journal.pone.0323761 (PMC12091834; doi:10.1371/journal.pone.0323761)
Supplement: S3 Table — (DOCX) [file pone.0323761.s003.docx]

**3. S3 Table.** The complete list of PD- and amphetamine-associated genes.

| **Item** | **Total Number** | **List** |
| --- | --- | --- |
| Parkinson  disease | 35 | EIF4G1, DNAJC6, PARK7, BST1, VPS13C, PINK1, DRD1, SLC6A3, ATXN2, GDNF, MAOB, SLC18A2, ATP6AP2, DDC, PLA2G6, FBXO7, CHCHD2, GBA, POLG, SYNJ1, SNCA, GIGYF2, LRRK2, MAPT, ATP13A2, UCHL1, GAK, VPS35, GSR, HTRA2, TH, PRKN, TBP, DRD2, IGF1R |
| Amphetamine | 63 | SLC6A3, SLC6A4, SLC6A2, FOS, TH, TAAR1, HTR2A, SLC18A2, PPP1R1B, DRD1, MAOB, DRD2, MAOA, HTR7, GRIA1, ADRB1, PDYN, ADRA1A, ADRA1B, GRM5, CARTPT, ADRA2A, GAD2, RGS4, CYP2D6, GRIN1, H3F3B, H3F3A, NGF, ADORA2A, ADCYAP1, KCNJ1, ATF4, STK11, TLR3, GJA1, CREM, FGF2, MAPK3, RASD1, WNT3, TMPRSS11D, SNCA, CAT, CSNK1E, F3, SOD1, DDC, DRD4, SLC18A1, OPRK1, OPRM1, HTR1A,, ADRA1D, ADRA2B, SLC6A9, DRD5, ADRA2C, NTSR1, NTSR2, FOSB, DRD3, HTR2C |
