# Supplementary material for: Amphetamine use and Parkinson’s disease: integration of artificial intelligence prediction, clinical corroboration, and mechanism of action analyses
Source: PLoS One. 2025 May 20;20(5):e0323761. doi: 10.1371/journal.pone.0323761 (PMC12091834; doi:10.1371/journal.pone.0323761)
Supplement: S4 Table — (DOCX) [file pone.0323761.s004.docx]

**4. S4 Table.** The full list enriched pathways for PD and amphetamine

| **Item** | **Total Number** | **Pathways** | **Pathway ID** |
| --- | --- | --- | --- |
| Parkinson  disease | 12 | Parkinson disease | hsa05012 |
|  |  | Cocaine addiction | hsa05030 |
|  |  | Amphetamine addiction | hsa05031 |
|  |  | Dopaminergic synapse | hsa04728 |
|  |  | Pathways of neurodegeneration - multiple diseases | hsa05022 |
|  |  | Alcoholism | hsa05034 |
|  |  | Tyrosine metabolism | hsa00350 |
|  |  | Phenylalanine metabolism | hsa00360 |
|  |  | Serotonergic synapse | hsa04726 |
|  |  | Tryptophan metabolism | hsa00380 |
|  |  | Mitophagy - animal | hsa04137 |
|  |  | Synaptic vesicle cycle | hsa04721 |
| Amphetamine | 22 | Neuroactive ligand-receptor interaction | hsa04080 |
|  |  | Cocaine addiction | hsa05030 |
|  |  | Amphetamine addiction | hsa05031 |
|  |  | Dopaminergic synapse | hsa04728 |
|  |  | Alcoholism | hsa05034 |
|  |  | Serotonergic synapse | hsa04726 |
|  |  | Calcium signaling pathway | hsa04020 |
|  |  | cAMP signaling pathway | hsa04024 |
|  |  | Gap junction | hsa04540 |
|  |  | Parkinson disease | hsa05012 |
|  |  | cGMP-PKG signaling pathway | hsa04022 |
|  |  | Synaptic vesicle cycle | hsa04721 |
|  |  | Circadian entrainment | hsa04713 |
|  |  | Adrenergic signaling in cardiomyocytes | hsa04261 |
|  |  | Long-term potentiation | hsa04720 |
|  |  | Pathways of neurodegeneration - multiple diseases | hsa05022 |
|  |  | Tyrosine metabolism | hsa00350 |
|  |  | Phenylalanine metabolism | hsa00360 |
|  |  | Tryptophan metabolism | hsa00380 |
|  |  | Vascular smooth muscle contraction | hsa04270 |
|  |  | Rap1 signaling pathway | hsa04015 |
|  |  | Salivary secretion | hsa04970 |
